# Trends in Hospitalisations Due to Respiratory Syncytial Virus in Swedish Children: A Nationwide Study 2010–2022

**DOI:** 10.1111/apa.70484

**Published:** 2026-02-24

**Authors:** Kristian Bolin, Karin Strandell, Andreas Palmborg, Sofie Gustafsson, Sven‐Arne Silfverdal

**Affiliations:** ^1^ Centre for health Governance, Department of Economics University of Gothenburg Gothenburg Sweden; ^2^ Department of Clinical Sciences, Paediatrics Umeå University Umeå Sweden; ^3^ Medical and Scientific Affairs Vaccines Pfizer AB Stockholm Sweden; ^4^ Department of Global Public Health, Karolinska Institutet, Stockholm, Sweden Pfizer AB Stockholm Sweden

**Keywords:** gestational age at birth, hospitalisation, RSV

## Abstract

**Aim:**

Respiratory syncytial virus (RSV) infection is a leading cause of paediatric hospitalisations. This study describes annual RSV‐hospitalisation burden of disease, by gestational week of delivery, age and sex among children aged 0–17 years of age in Sweden, for the period 2010/11–2021/22.

**Methods:**

Employing total population Swedish register data pertaining to hospitalisations due to RSV for the period 2010–2022.

**Results:**

Over the study period a total of 26 772 children were hospitalised due to RSV. Most of the patients, 63.2%, were aged < 6 months and 39.9% were aged < 3 months. Among patients aged < 3 months, 88.5% were born at full term. The average number of hospital days increased with lower gestational age at birth for all age groups, except for patients aged 5‐ ≤ 17 years of age. Estimated incident rate ratios (IRR) comparing patients born at full term to patients born extremely preterm ranged between 2.43 and 1.75, respectively, for patients aged 0 < 3 months and 1 < 2 years of age.

**Conclusions:**

Paediatric RSV hospitalisations mostly occur in the youngest infants, and most are born at term. Gestational age at birth and age at admission are both negatively related to length of stay.

## Summary

- Paediatric RSV hospitalisations mostly occur in the youngest infants, and most were born at term.
- The seasonal pattern of child hospitalisations due to RSV was disrupted by the COVID‐19 pandemic.
- Gestational age at birth and age at admission were negatively related to length of hospital stay.

**Abbreviations**
COVID: Corona virus disease
ICD10: 10th revision of the international classification of diseases
PCR: Polymerase chain reaction
RSV: Respiratory syncytial virus
wGA: weeks of gestational age

## 1 Introduction

Respiratory syncytial virus (RSV) infection is a leading cause of paediatric hospitalisations [1, 2]. Studies have shown that pre‐term birth, age, male gender and concurrent medical conditions affect the risk for hospitalisation due to RSV [1, 2, 3, 4, 5, 6, 7, 8, 9, 10]. Fewer studies have examined the relationship between gestational age at birth, age at hospital admission and the *length* of hospital stay once admitted [11, 12, 13].

Until 2023 palivizumab, a short acting monoclonal antibody has been the only preventive treatment against RSV disease for children. According to Swedish guidelines during the study period, this treatment has only been available to infants born before gestational week 26, and children with severe lung‐ or cardiac disease [14]. In recent years novel interventions for prevention and treatment of RSV in children have become available or are in late‐stage clinical development. These interventions include vaccines for maternal immunisation, i.e., vaccination of the pregnant woman to protect the newborn child [15], paediatric vaccination [16], monoclonal antibodies and antivirals [17]. Both maternal vaccination and long‐acting monoclonal antibodies are in clinical use. Vaccinations for children and antiviral treatment against RSV are not available. Optimal design of policy initiatives, such as vaccination programmes, requires epidemiological information ideally generated from total‐population based studies employing longitudinal individual‐level health‐care data.

The aim of this study is to describe RSV burden of disease (hospitalisations) by weeks of gestational age at birth (wGA), age and sex among hospitalised children aged 0–17 years of age in Sweden in‐between the RSV seasons 2010/11–2022/2.

## 2 Data and Methods

This study exploited Swedish total‐population longitudinal individual‐level register data on RSV hospitalisation, mortality and gestational age at birth for the period from and including 2010 to and including 2022. More specifically, the dataset employed in the analyses was constructed in two steps. *First*, the study population was defined as all patients who had at least one hospitalisation due to RSV (≤ 17 years of age at that time) registered in the Swedish Patient Register (as main diagnosis) from and including 2010 to and including 2022, where RSV was defined as at least one of the following ICD10 codes as main diagnosis: B97.4, J21.0, J12.1, J20.5. *Second*, for these patients information about (a) mortality and (b) gestational age at birth was collected from the Swedish Cause of Death Register and the Swedish Medical Birth Register, respectively. Analyses pertaining to gestational age at birth include patients *born* from and including 2010 to and including 2022.

### 2.1 Data Analysis

All analyses pertain to hospitalisations due to RSV among children (≤ 17 years of age at the date of hospital admission) for the entire period 2010–2022. The population is described by calculating the number of patients and the number of hospitalisations for three different partitions of the population. More specifically, we calculated, *first*, the number of patients over the entire period, separately for the age groups < 3 months, 3 < 6 months, 6 < 12 months, 1 < 2 years, 2 < 5 years and 5 ≤ 17 years, at hospital admission, *and*, for each group, by gestational age at birth (full term, ≥ 37 weeks; late preterm, 32–36 weeks; early preterm, 28–31 weeks; extreme preterm, ≤ 27 weeks) and sex.

*Second*, the *annual* number of patients hospitalised due to RSV by the age groups, gestational age at birth and sex were calculated for each annual period from, and including, July 2010, to and including December 2022. *Third*, the annual (July 2010—June 2022) number of hospitalisations due to RSV per age group was calculated.

The following outcomes pertaining to hospital admissions were calculated: by the aforementioned age groups (a) shares of hospitalisations involving same‐day discharge, share of hospitalisations with a duration longer than 7 days, and average number of hospital days per admission, respectively, and by gestational age at birth, (b) average number of hospital days, and (c) incidence rate ratios pertaining to length of hospital.

### 2.2 Statistical Methods

Incidence rate ratios pertaining to length of hospital stay were estimated by negative binomial regressions separately for each of the previously mentioned age groups, comparing gestational ages at birth. The variables employed are described together with the statistical specifications in the Appendix.

### 2.3 Standard Protocol Approvals, Registrations and Patient Consents

This study has been approved by the Swedish Ethical Review Authority.

## 3 Results

**FIGURE 1:**
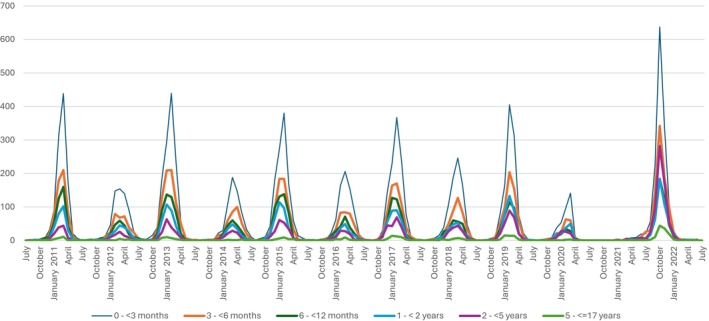
Number of hospitalisations due to RSV, per age group and year.

Descriptive statistics for the studied population are reported in (Tables 1 and 2 and Figure 1). The number of unique patients who received inpatient care due to RSV is reported for the total population (a) by age groups, cross‐tabulated against the population partitioned by age groups, sex, gestational age at birth, respectively (Table 1), and (b) by year, 2010/11–2022/22 (July to June the subsequent year) cross‐tabulated against the aforementioned categories (Table 2). The corresponding annual numbers of hospitalisations per age group are illustrated in (Figure 1).

**TABLE 1:** Total number of patients hospitalised due to RSV per age group, and by gestational age at birth, and clinical outcomes, 2010–2022.

	0–< 3 months	3–< 6 months	6–< 12 months	Age 1 < 2	Age 2 < 5	Age 5 ≤ 17
Total number of hospitalised patients*	10691	6225	4041	3324	2491	381
Total, born 2010 or after	10410	5664	3625	2869	2124	189
Gestational age at birth (share of total population, %)**						
Full term (≥ 37 wGA)	9462 (90.9%)	4797 (84.7%)	3029 (83.6%)	2382 (83.0%)	1662 (78.2%)	144 (76.2%)
Late preterm (32–36 wGA)	892 (8.6%)	678 (12.0%)	384 (10.6%)	296 (10.3%)	226 (10.6%)	25 (13.2%)
Early preterm (28–31 wGA)	50 (0.5%)	134 (2.4%)	155 (4.3%)	96 (3.3%)	92 (4.3%)	10 (5.3%)
Extreme preterm (≤ 27 wGA)	6 (0.1%)	55 (1.0%)	57 (1.6%)	95 (3.3%)	144 (6.8%)	10 (5.3%)
Sex						
Male	5790 (54.2%)	3624 (58.2%)	2421 (59.9%)	1823 (54.8%)	1254 (50.3%)	174 (45.7%)
Female	4901 (45.8%)	2601 (41.8%)	1620 (40.1%)	1501 (45.2%)	1237 (49.7%)	207 (54.3%)

* Some patients have been hospitalised at least two times, at different ages. Thus, the sum of patients in the first row > 26772.

** Information about gestational age at birth was only available for patients after 2009. Thus, the reported shares are in relation to this population.

**TABLE 2:** Number of patients with at least one hospitalisation due to RSV, per age group, sex and gestational age at birth. Number of unique children (per partitioning and year* ^)^ **.

	2010/11	2011/12	2012/13	2013/14	2014/15	2015/16	2016/17	2017/18	2018/19	2019/20	2020/21	2021/22
Number of hospitalised patients***	2347	1314	2864	1310	2590	1457	2550	1717	2502	822	109	3804
Total, born 2010 or after	2050	1252	2792	1297	2564	1447	2529	1705	2486	822	107	3794
Age												
0–< 3 months	1009 (43.0%)	562 (42.8%)	1182 (41.3%)	550 (42.0%)	1035 (40.0%)	654 (44.9%)	1012 (39.7%)	739 (43.0%)	981 (39.2%)	320 (38.9%)	41 (37.6%)	1332 (35.0%)
3–< 6 months	573 (24.4%)	312 (23.7%)	710 (24.8%)	322 (24.6%)	583 (22.5%)	336 (23.1%)	578 (22.7%)	389 (22.7%)	554 (22.1%)	177 (21.5%)	19 (17.4%)	824 (21.7%)
6–< 12 months	384 (16.4%)	196 (14.9%)	469 (16.4%)	197 (15.0%)	442 (17.1%)	212 (14.6%)	401 (15.7%)	235 (13.7%)	335 (13.4%)	102 (12.4%)	08 (7.3%)	459 (12.1%)
Age 1–< 2	252 (10.7%)	157 (11.9%)	316 (11.0%)	142 (10.8%)	328 (12.7%)	143 (9.8%)	309 (12.1%)	191 (11.1%)	339 (13.5%)	131 (15.9%)	17 (15.6%)	442 (11.6%)
Age 2–< 5	110 (4.7%)	76 (5.8%)	161 (5.6%)	91 (6.9%)	175 (6.8%)	96 (6.6%)	209 (8.2%)	140 (8.2%)	245 (9.8%)	86 (10.5%)	21 (19.3%)	635 (16.7%)
Age 5–≤ 17	19 (0.8%)	11 (0.8%)	26 (0.9%)	08 (0.6%)	27 (1.0%)	16 (1.1%)	41 (1.6%)	23 (1.3%)	48 (1.9%)	06 (0.7%)	03 (2.8%)	112 (2.9%)
**Sex**												
Male	1370 (58.4%)	730 (55.6%)	1665 (58.1%)	732 (55.9%)	1374 (53.1%)	808 (55.5%)	1427 (56.0%)	959 (55.9%)	1360 (54.4%)	431 (52.4%)	56 (51.4%)	2061 (54.2%)
Female	977 (41.6%)	581 (44.2%)	1199 (41.9%)	578 (44.1%)	1211 (46.8%)	647 (44.4%)	1120 (43.9%)	758 (44.1%)	1142 (45.6%)	391 (47.6%)	53 (48.6%)	1738 (45.7%)
**Gestational age at birth** ****												
Full term (≥ 37 wGA)	1746 (85.2%)	1054 (84.2%)	2339 (83.8%)	1099 (84.7%)	2117 (82.6%)	1185 (81.9%)	2094 (82.8%)	1447 (84.9%)	2096 (84.3%)	700 (85.2%)	97 (90.7%)	3238 (85.3%)
Late preterm (32–36 wGA)	221 (10.8%)	140 (11.2%)	280 (10.0%)	118 (9.1%)	266 (10.4%)	177 (12.2%)	282 (11.2%)	170 (10.0%)	255 (10.3%)	83 (10.1%)	04 (3.7%)	329 (8.7%)
Early preterm (28–31 wGA)	35 (1.7%)	28 (2.2%)	74 (2.7%)	32 (2.5%)	75 (2.9%)	34 (2.3%)	53 (2.1%)	22 (1.3%)	55 (2.2%)	12 (1.5%)	04 (3.7%)	90 (2.4%)
Extreme preterm (≤ 27 wGA)	11 (0.5%)	07 (0.6%)	38 (1.4%)	17 (1.3%)	40 (1.6%)	23 (1.6%)	50 (2.0%)	34 (2.0%)	43 (1.7%)	15 (1.8%)	02 (1.9%)	80 (2.1%)

* From and including July year n to and including June year *n* + 1.

** Note, patients may have been hospitalised in more than one period. Therefore, and because of the truncation mentioned in*, the figures reported are not perfectly comparable to the figures reported in Table 1.

*** 95% CI for the annual average total number of hospitalised patients prior to the COVID‐19 pandemic, that is 2010/11–2019/20: 42–3065. Thus, the number of post‐pandemic hospitalised patient, 3804, differs significantly from the expected annual number of hospitalised patients prior to the pandemic.

**** Includes only patients born in 2010 or after.

Over the study period a total of 26 772 children were hospitalised due to RSV (29332 hospital admissions). A majority, 16916 (63.2%) patients were below 6 months of age and 10691 (39.9%) were aged < 3 months. Among patients < 3 months of age the absolute majority, 9462 (90.9%), were born full term (≥ 37 wGA), 892 (8.6%) were born late preterm (32–36 wGA). Further, for all age groups, except 5– ≤ 17 years of age, more males than females were hospitalised (Table 1). The hospitalisation time‐patterns among the different age groups, males and females and gestational age groups are reported in Table 2. Although more males than females were hospitalised each seasonal period, the difference was largest in 2010/11: about 40% more males than females were hospitalised. During the seasons 2019/20 and 2020/21 (beginning of the COVID‐19 pandemic), there were very few cases hospitalised, with a very small sex difference. During the following season 2021–2022, again more males than females were hospitalised.

The highest number of hospitalisations, 3804 in total, was observed during the 2021/2022 season while, in contrast, the season 2020/21 showed the lowest number (109 hospitalisations; Table 2). On average 1904 children required hospitalisation each season. The youngest age group (< 3 month of age) constituted 43% of the patients in 2010/11. This share reached a maximum for the period in 2015/16 with almost 45% of the patients younger than 3 months. In 2021/22 the corresponding figure had declined to 35%. Over the entire period, the share of children hospitalised due to RSV at ages 2 < 5 and 5 ≤ 17, respectively, increased by a factor between 3 and 4, while the corresponding shares pertaining to children aged 3 < 6 and 6 < 12 months, respectively, decreased from about 24% and 16% in 2010/11 to 22% and 12% in 2021/22. In comparison, the changes in share of patients at different gestational age at birth over the observed time periods were small. Perhaps the most noteworthy is the increase of extreme preterm children from 0.5% in 2010/11 to 2.1% in 2021/22.

The seasonal pattern of hospitalisations depicted in Figure 1 reveals a pattern in which a season with a high hospitalisation frequency is followed by a season with a lower hospitalisation frequency, with no peak at all in 2020/21 followed by a very strong increase in 2021/22 in all age groups.

The number of all‐cause deaths, 23, occurring within 30 days of healthcare contact due to RSV occurred during the study period corresponding to a mortality rate of 0.09% (not reported in table). Distribution of hospital days and length of hospital stay are reported in Table 3. The average numbers of hospital days (95% CI) by age group and by gestational age at birth are reported in the upper half of Table 3. The average number of hospital days ranged between 3.4 and 4.4 for the different age groups. The length of stay age pattern is u‐shaped: among the youngest children, < 3 months of age, and among children 5 < 17 years of age, 19.9% and 23.6%, respectively, of the hospitalisations had a duration ≥ 7 days, while a smaller fraction of the hospitalisation events among children in the intermediate age groups required ≥ 7 days.

**TABLE 3:** Outcomes—Distribution of hospital days, and average number of hospital days and IRRs pertaining to length of stay, respectively, per age group and by gestational age at birth. Incidence rate ratios, IRR (95% CI) were estimated by negative binomial specifications containing a dummy variable indicating the compared groups. Regressions were performed separately for each age group.

Age—distribution of hospital days						
	< 3 months	3–< 6 months	6–< 12 months	1–< 2 years	2–< 5 years	5–≤ 17 years
Same day discharge	2.7%	3.6%	3.6%	4.0%	5.0%	5.8%
LOS ≥ 7 days	19.9%	13.4%	13.3%	10.4%	12.0%	23.6%
Average number of hospital days	4.4	3.8	3.7	3.4	3.4	4.4
Average length of stay (95% CI*) per age group and gestational age at birth						
Gestational age at birth	< 3 months	3–< 6 months	6–< 12 months	1–< 2 years	2–< 5 years	5–≤ 17 years
wGA = full term (comparator)	4.27 (4.21–4.32) (10387)	3.49 (3.42–3.56) (5058)	3.52 (3.42–3.62) (3183)	3.29 (3.18–3.39) (2502)	2.99 (2.85–3.12) (1771)	3.59 (3.06–4.11) (160)
wGA = late preterm	**5.35** (5.09–5.61) (1023)	**4.61** (4.31–4.92) (746)	**4.13** (3.81–4.45) (419)	**3.74** (3.44–4.03) (325)	**3.75** (3.36–4.15) (251)	4.92 (2.93–6.91) (26)
wGA = early preterm	**8.41** (5.88–10.94) (68)	**5.80** (5.11–6.49) (161)	**5.08** (4.42–5.74) (170)	**4.02** (3.47–4.57) (105)	**4.14** (3.45–4.83) (104)	3.14 (2.00–4.28) [14]
wGA = extreme preterm	**10.38** (5.15–15.60) (08)	**6.73** (5.44–8.01) (66)	**6.68** (4.11–9.26) (63)	**5.75** (4.94–6.56) (105)	**5.49** (4.83–6.15) (169)	4.00 (1.52–6.48) [12]
Length of stay (IRRs as compared to full term)						
wGA = full term (comparator)						
wGA = late preterm	**1.25** (1.20–1.31)	**1.32** (1.25–1.39)	**1.17** (1.09–1.26)	**1.14** (1.05–1.24)	**1.26** (1.13–1.39)	1.37 (0.95–1.98)
wGA = early preterm	**1.97** (1.71–2.27)	**1.66** (1.51–1.83)	**1.44** (1.30–1.60)	**1.22** (1.07–1.40)	**1.39** (1.19–1.61)	0.88 (0.53–1.44)
wGA = extreme preterm	**2.43** (1.64–3.61)	**1.93** (1.67–2.23)	**1.90** (1.61–2.23)	**1.75** (1.54–1.99)	**1.84** (1.64–2.06)	1.12 (0.66–1.88)

*Note:* Bold indicate that the average is significantly different from its comparator. Each category of gestational age at birth is compared to full term. Significance assessed by *t*‐test of difference in means.

Abbreviations: LOS, length of stay; wGA, week of gestational age.

The average length of stay by age group and gestational age at birth varied between 10.38 (children aged < 3 months and born extremely preterm) and 3.74 (children aged 1 < 2 years of age and born late preterm). The comparisons between children born at full term and children born preterm reveal a clear pattern: for each age group, except for the oldest age group, the more prematurely born, the longer is the length of hospital stay (significance assessed from non‐overlapping CIs).

Incidence rate ratios pertaining to length of hospitalisation stay (days) are reported in the lower half of Table 3, again, per age group, and per gestational age at birth, respectively. Children born prematurely were hospitalised for longer durations compared to children born at full term. More specifically, gestational age at birth decreased the length of stay except for those in the age group 5 – ≤ 17 years of age. The effect of being born prematurely on the length of hospital stay is consistently higher than 1.14 (late preterm 1–< 2 years vs. full term), and considerably higher for most compared groups. In particular, patients born extremely premature and aged < 3 months were estimated to have 243% longer hospitalisations than those (in the same age group) born at full term.

## 4 Discussion

In this study, exploiting Swedish comprehensive nationwide patient‐level data on hospitalisations for the years 2010–2022, we identified 26 772 children (≤ 17 years of age) who were hospitalised due to RSV. Among those, a majority were younger than 6 months of age and born at full term.

The estimated within season and between season pattern showed a cyclical variation with maximum hospitalisation frequencies accruing during the winter and a high‐frequency season followed by a low‐frequency season.

Although the COVID‐19 pandemic in Sweden was initially treated differently than in many other countries [18, 19] the pandemic did disrupt the cyclical pattern in as much as the low‐frequency season of 2020/21 was followed by a season with an even lower hospitalisation frequency. The seasonal pattern observed in our data has been previously observed and reported by, for example, [11]. In the last season of this study, an increase in RSV hospitalisations was seen in all age groups. Previous studies [11, 20, 21, 22] have shown similar results, but not to our knowledge in children over 5 years of age. This increase in RSV hospitalisations in 2021/22 may be due to the low levels of RSV circulating during the previous seasons and thereby affecting immunity in several ways: such as reduced levels of maternally derived RSV‐antibodies, postponed primary RSV infection in toddlers, and waning immunity in older children due to no reinfection with RSV. A cohort study from Denmark showed that the hospitalisation rate and need for mechanical ventilation due to RSV was twice as high when RSV resurfaced after non‐pharmaceutical interventions due to the COVID‐19 pandemic were lifted compared to pre‐pandemic years. The increase was highest among children aged 2–5 years and older children without risk factors (age under 3 months and comorbidities) had other reasons than classical bronchiolitis that caused the need for mechanical ventilation [23]. Implementing various methods to protect the youngest and those most at risk for being hospitalised due to RSV, such as maternal vaccination and long‐acting monoclonal antibodies, stress the importance of monitoring the risk of RSV hospitalisation in older children.

Most children hospitalised due to RSV were < 6 months of age and born at full term. Being born more prematurely was consistently associated with longer hospitalisation, except among patients aged 5 – ≤ 17 years of age at admission. For each age group, except 5 – ≤ 17 years of age, the effect on length of hospital stay decreased successively by gestational age at birth. This suggests that being born prematurely has persisting health effects at least until 5 years of age, and that this effect is larger the younger the patient is at admission.

The results reported in this study suggest that the scope for RSV preventive interventions is considerable. More specifically, the potential benefits of such interventions are larger the younger the target population, since the frequency, and therefore the absolute risk, of RSV associated hospitalisations decreases with age (at admission). The implementation of such interventions should be followed in real life clinical practice to understand whether their effectiveness aligns with results demonstrated in clinical trials [15, 17]. The importance of gestational age at birth in this regard is not due to the frequencies of hospitalisations, since an absolute majority of children are born at full term. Rather, the significance of gestational age at birth is the risk of hospitalisation due to RSV. Even though we did not estimate the risks of being hospitalised comparing age and gestational age at birth groups, a crude comparison of our data with the reported number of births by gestational age at birth (the Swedish National Board of Health and Welfare) suggests that these risks are higher the lower the gestational age at birth. For example, during the studied period about 3500 children were born extremely preterm and 367 of these were hospitalised due to RSV, while the corresponding figures for children born at full term were about 1400 000 and 21 500, implying that the absolute risk for being hospitalised due to RSV is more than 6 times higher for children born extremely preterm. Extremely premature children are frequently provided with prophylactic treatment with monoclonal antibodies, without which the observed absolute risk difference would have been even larger. The same comparisons between children being born late and early preterm, respectively, and children born at full term show that the former groups face risks of hospitalisation due to RSV that are about 3 and 6 times as high as children born at full term.

## 5 Strengths and Limitations

The strength of this study is that data employed is of high quality and covers the entire population. The following limitations are noteworthy. First, the data analyses are based on ICD‐diagnoses. No laboratory register‐data were used to confirm infections. RSV in the youngest children has always been a serious condition why the testing and diagnostic is a priority, and we claim that this practice has not changed during the study period. Second, the use of the more sensitive multiplex PCR increased from around 2013/14 as well as the testing frequency increased furthermore during the pandemic which may have led to more identified and reported RSV cases during and after the pandemic compared to the seasons before the pandemic, especially in older children [24]. Third, confounding variables, such as breastfeeding exposure, socioeconomic status and comorbidities, that are not captured in the dataset may influence the findings. Information on comorbidities would be of particular interest in the oldest age groups. First, the share of hospitalised patients in the age group 5 < 17 who were hospitalised ≥ 7 days is considerably higher than in all other age groups. Second, we found no significant relationship between gestational age at birth and length of stay in this age group. Thus, it is plausible that the relative high share of patient observed being hospitalised ≥ 7 days reflects underlying health conditions. Indeed, additional estimates (see Appendix S1) show that respiratory disease is positively related to length of stay among the oldest children. Moreover, for the age groups 1 < 2 and 2 < 5 years of age both cardiovascular disease and perinatal conditions, respectively, were found to be positively associated with length of stay.

## 6 Conclusions

Four main conclusions can be drawn:

1. Paediatric RSV hospitalisations mostly occur in the youngest infants, and most are born at term.
2. The seasonal pattern of child hospitalisations due to RSV was disrupted by the COVID pandemic. During the years preceding the COVID‐19 years between 2000 and 3000 children were hospitalised during the high‐frequency seasons and about 1000–1500 during the low frequency seasons. These figures stand in sharp contrast to the initial COVID‐19 pandemic years during which about 100 children were hospitalised.
3. Gestational age at birth is negatively related to length of hospital stay. The effect of being born prematurely is strong in all age groups, except among the oldest children.
4. More male than female children are hospitalised due to RSV. The difference varies over the years reported. During the COVID‐19 years this difference disappeared but reappeared after the pandemic.

## Author Contributions

Kristian Bolin: Major role in design of study and acquisition of data. Drafted the manuscript for intellectual content. Karin Strandell: Major role in design of study. Drafted the manuscript for intellectual content. Andreas Palmborg: Major role in design of study. Drafted the manuscript for intellectual content. Sofie Gustafsson: Major role in design of study. Drafted the manuscript for intellectual content. Sven‐Arne Silfverdal: Major role in design of study. Drafted the manuscript for intellectual content. UCB: Design of study; interpreted the data; revised the manuscript for intellectual content.

## Funding

This work was supported by Pfizer Sweden.

## Conflicts of Interest

Professor Kristian Bolin reports funding from Pfizer. Karin Strandell declares no conflicts of interest. Andreas Palmborg, employee of Pfizer and holds stock and stock options in Pfizer. Sofie Gustafsson, employee of Pfizer and holds stock and stock options in Pfizer. Sven‐Arne Silfverdal has received honoraria as member of a Pfizer advisory board 2023 and 2025 on pneumococcal vaccines, and as member of MSD and SanofiPasteur ad boards, respectively, on childhood vaccines and RSV prevention. This study was sponsored by Pfizer.

## Supporting information


**Appendix S1:** Supporting Information.

## Data Availability

The data that support the findings of this study are available on request from the corresponding author. The data are not publicly available due to privacy or ethical restrictions.

## Appendix A

### Statistical Specification

The length of hospital stay (number of days) was estimated separately for the age groups (see below), and by (a) comparing length of hospitalisation due to RSV occurring among patients of each non‐full gestational‐age‐at‐birth group to length of hospitalisation among patients who were born at full term, and (b) comparing length of hospitalisation among patients with a concurrent comorbidity to length of hospitalisation among patients without concurrent comorbidity.

Formally, the following general specifications were used:

$$d_{\mathrm{ij}}={\text{constant}}_{\mathrm{ij}}+\beta x_{\mathrm{ij}},$$

where $d_{\mathrm{ij}}\geq 0$ is the length of stay (number of days) for age group *i* and gestational‐age‐at‐birth. So, *i* = age groups (< 3 months, 3 < 6 months, 6 < 12 months, 1 < 2 years, 2 < 5 years and 5 ≤ 17 years, at hospital admission}, and j={ full term vs. late preterm, full term vs. late preterm, full term vs. early preterm, vs. full term vs. extreme preterm}. Then, *x*_ij_ is a dummy variable equal to 1 for observations in age group *i* and non‐comparison group *j* (i.e., late preterm, early preterm, extreme preterm). This means that 18 equations were estimated.
