# Supplementary material for: Trends in Hospitalisations Due to Respiratory Syncytial Virus in Swedish Children: A Nationwide Study 2010–2022
Source: Acta Paediatr. 2026 Feb 24;115(6):1258–65. doi: 10.1111/apa.70484 (PMC13159787; doi:10.1111/apa.70484)
Supplement: Supplementary file 1 — Appendix S1: Supporting Information. [file APA-115-1258-s001.docx]

| Table A1. Outcomes – relative risks (95% CI) for hospitalization (conditional on healthcare contact due to RSV, according to age group, and gestational age at birth and comorbidities | | | | | | |
| --- | --- | --- | --- | --- | --- | --- |
|  | 0-<3 months | 3-<6 months | 6 to <12 months | Age 1 - <2  vs < 1 year of age | Age 2 - <5  vs < 1 year of age | Age 5 - < 17  vs < 1 year of age |
| **Gestational age at birth** | | | | | | |
| Full term (≥37 wGA) | - | - | - | - | - | - |
| Late preterm (32-36 wGA) | **1,14** (1,11 -1,18) | 0,94 (0,89 -0,98) | 0,87 (0,82 -0,93) | 1,04 (0,98 -1,11) | **1,10** (1,03 -1,18) | 1,00 (0,79 -1,27) |
| Early preterm (28-31 wGA) | **1,26** (1,14 -1,41) | **1,13** (1,04 -1,22) | 1,02 (0,93 -1,11) | **1,23** (1,12 -1,35) | **1,24** (1,13 -1,35) | 1,23 (0,96 -1,57) |
| Extreme preterm (≤27 wGA) | **1,26** (0,93 -1,72) | **1,26** (1,13 -1,40) | 1,06 (0,92 -1,22) | 1,09 (0,98 -1,22) | **1,14** (1,05 -1,24) | **1,35** (1,09 -1,68) |
| **Comorbidities*** | | | | | | |
| Asthma vs no comorbidity | 1,07 (0,97 -1,17) | **1,12** (1,03 -1,21) | **1,22** (1,15 -1,29) | **1,09** (1,04 -1,14) | 1,03 (0,98 -1,08) | 1,01 (0,90 -1,13) |
| Cancer vs no comorbidity | 1,04 (0,81 -1,34) | 0,94 (0,73 -1,23) | 1,03 (0,86 -1,22) | 1,15 (1,00 -1,32) | 1,06 (0,96 -1,18) | 0,98 (0,84 -1,15) |
| Cardiovascular disease vs no comorbidity | **1,38** (1,17 -1,63) | 1,15 (0,90 -1,47) | **1,23** (1,03 -1,47) | **1,16** (1,02 -1,31) | **1,11** (1,01 -1,21) | 1,11 (0,96 -1,30) |
| Chronic respiratory disease (not Asthma) vs no comorbidity | **1,11** (1,09 -1,14) | **1,16** (1,12 -1,20) | **1,10** (1,06 -1,14) | **1,08** (1,04 -1,12) | **1,11** (1,06 -1,16) | 1,09 (0,93 -1,26) |
| Perinatal conditions vs no comorbidity | **1,12** (1,07 -1,18) | **1,28** (1,21 -1,34) | **1,15** (1,08 -1,23) | 1,03 (0,96 -1,10) | 1,06 (1,00 -1,13) | **1,24** (1,07 -1,43) |
| * Information about hospital visits, or hospitalizations, prior to or at the same date as the first registered RSV was used to identify patients who had concurrent comorbidities at the time of health-care contacts due to RSV. Comorbidities are defined by the following ICD10 codes. Asthma: J45; Cancer: C00-C99; Cardiovascular disease: I40, I42, I50, I52, I60-I69; Chronic respiratory disease: J except J45; Disorders related to short gestation and low birth weight: P07. | | | | | | |

Table A2. Table A2 complements Table 3 in the main manuscript.

| Table A2. Outcomes – length of hospital stay, according to age group, and gestational age at birth and comorbidities. Incidence rate ratios, IRR (95 % CI) estimated by negative binomial specifications containing a dummy variable indicating the compared groups. Regression were performed separately for each age group. | | | | | | |
| --- | --- | --- | --- | --- | --- | --- |
|  | 0-<3 months | 3-<6 months | 6 to <12 months | Age 1 - <2  vs < 1 year of age | Age 2 - <5  vs < 1 year of age | Age 5 - < 17  vs < 1 year of age |
| **Comorbidities (IRRs as compared to no comorbidity)*** | | | | | | |
| Asthma vs no comorbidity | 0,90 (0,80 -1,01) | 1,04 (0,95 -1,14) | 1,17 (1,09 -1,25) | **1,13** (1,06 -1,20) | **1,15** (1,08 -1,23) | 1,01 (0,84 -1,20) |
| Cancer vs no comorbidity | 1,21 (0,90 -1,62) | 1,13 (0,87 -1,47) | 1,06 (0,87 -1,29) | **1,33** (1,10 -1,61) | **1,52** (1,30 -1,77) | 0,93 (0,73 -1,18) |
| Cardiovascular disease vs no comorbidity | 1,01 (0,72 -1,42) | **2,00** (1,56 -2,56) | **1,33** (1,06 -1,65) | **1,38** (1,16 -1,64) | **1,56** (1,35 -1,80) | 1,01 (0,78 -1,32) |
| Chronic respiratory disease (not Asthma) vs no comorbidity | 0,96 (0,94 -0,99) | 1,01 (0,97 -1,05) | **1,15** (1,10 -1,21) | **1,20** (1,14 -1,26) | **1,27** (1,19 -1,35) | **1,41** (1,11 -1,77) |
| Perinatal conditions vs no comorbidity | **1,36** (1,27 -1,44) | **1,40** (1,31 -1,48) | **1,32** (1,23 -1,43) | **1,36** (1,25 -1,47) | **1,43** (1,31 -1,56) | 1,02 (0,76 -1,38) |
| * Information about hospital visits, or hospitalizations, prior to or at the same date as the first registered RSV was used to identify patients who had concurrent comorbidities at the time of health-care contacts due to RSV. Comorbidities are defined by the following ICD10 codes. Asthma: J45; Cancer: C00-C99; Cardiovascular disease: I40, I42, I50, I52, I60-I69; Chronic respiratory disease: J except J45; Disorders related to short gestation and low birth weight: P07. | | | | | | |
